# Full-Mouth Digital Dental Rehabilitation Under General Anesthesia in a Post-Treatment Intraoral Squamous Cell Carcinoma Patient: A Case Report

**DOI:** 10.3390/healthcare13080940

**Published:** 2025-04-19

**Authors:** Cindy Batisse, Nada El Osta, Pierre-Yves Cousson

**Affiliations:** 1 Department of Prosthodontics, UFR d’Odontologie, Clinical Odontology Research Center (CROC), University of Clermont Auvergne, F-63000 Clermont-Ferrand, France; cindy.lance@uca.fr; 2 CHU Clermont-Ferrand, Odontology Department, F-63000 Clermont-Ferrand, France; p-yves.cousson@uca.fr; 3 Department of Odontology, UFR d’Odontologie, Clinical Odontology Research Center (CROC), University of Clermont Auvergne, F-63000 Clermont-Ferrand, France

**Keywords:** full-mouth oral rehabilitation, CAD-CAM, prosthetic dentistry, mandibular symphyseal squamous cell carcinoma, dental treatment

## Abstract

**Background:** The treatment of head and neck cancer primarily involves surgical tumor removal combined with radiotherapy and/or chemotherapy. It often leads to significant side effects, impacting the anatomical structures of the oral cavity and resulting in major functional, esthetic, and socio-relational alterations. **Case presentation:** This clinical report aims to demonstrate the effectiveness of a hospital-based approach incorporating general anesthesia (GA) and computer-aided design and manufacturing (CAD/CAM) technology in the oral rehabilitation of a 58-year-old woman in remission from intraoral squamous cell carcinoma of the mandibular symphysis. The patient presented with oral pain, radiation-induced caries, reduced occlusal vertical dimension, and severely compromised teeth. **Treatment Approach:** The treatment plan included the removal of two non-restorable teeth, root canal treatment for the remaining teeth, and the placement of ceramic crowns and a partial removable prosthesis. Due to the complexity of the case and the patient’s limitations, the treatment was performed under GA, allowing for a staged approach. Digital technologies, including intraoral scanning and CAD-CAM, enhanced precision and patient comfort. This approach facilitated tooth preservation and minimized the number of extractions while achieving satisfactory functional and esthetic outcomes. **Conclusion:** The case highlights the value of GA and digital techniques in managing special-needs patients with a history of irradiated head and neck cancer.

## 1. Introduction

Intraoral squamous cell carcinoma (SCC) is a malignant epithelial tumor that has the potential to invade the underlying bone, compromising both the structural and functional integrity of the maxilla or mandible. Treatment of SCC typically involves a combination of surgical resection, radiotherapy, and chemotherapy. While these interventions are effective in managing the tumor, they often result in side effects that severely impact the anatomical structures of the oral cavity, leading to functional impairments [1,2,3,4], changes in appearance, and a reduction in quality of life [5,6,7].

Surgical resection of SCC often results in substantial tissue loss, altering the anatomy of the oral cavity. In cases involving the mandible, such alterations can cause lateral deviation, occlusal problems, and salivary incontinence, with sensory disturbances due to nerve damage. These functional changes can also affect esthetics outcomes, often exacerbated by rapid weight loss [6]. In addition to these physical changes, patients may suffer from tissue support loss, scarring, and facial asymmetry, contributing to identity disturbances and social withdrawal, which affect their quality of life [5]. Radiotherapy induces irreversible changes in oral tissues, particularly in salivary glands, leading to hypofunction and xerostomia. This, in turn, increases the risk of dental caries and radiation-induced fibrosis, which can result in trismus [3]. Radiation doses also increase the risk of osteoradionecrosis (ORN) [7,8,9]. Chemotherapy further complicates the recovery process by inducing mucositis, immunosuppression, and prolonged healing times, which exacerbate the challenges in oral rehabilitation [10].

The literature emphasizes the importance of preventive care before and during cancer treatments. Following cancer treatment, the effective restoration of caries is essential to prevent disease progression and reduce the risk of tooth loss [11]. Preserving as many teeth as possible improves patients’ overall quality of life.

Post-cancer oral rehabilitation requires a personalized approach to restore function while considering anatomical limitations. Implant treatments can be prioritized in rehabilitation due to its positive impact on mastication and overall quality of life; however, its success is dependent on radiation dose and bone quality. Studies indicate that implant survival rates decline in irradiated bone, particularly when radiation doses exceed 50–60 Gy [12,13,14,15]. Therefore, a multidisciplinary approach from the time of cancer diagnosis is essential, allowing for pre-surgical implant planning when feasible to optimize rehabilitation outcomes [16]. Early decision-making regarding potential implant placement is vital for long-term prosthodontic success in head and neck cancer patients. When implants are not feasible, alternative solutions include removable partial dentures and fixed dental prostheses. Removable partial dentures are practical in irradiated patients, as they avoid additional surgical interventions and adapt to compromised bone conditions. Fixed dental prostheses offer improved function and esthetics, but their success is dependent on the quality of the remaining dentition [10].

Advancements in digital dentistry, such as computer-aided design and computer-aided manufacturing (CAD/CAM) technology, have revolutionized prosthetic rehabilitation. CAD/CAM-fabricated crowns and RPDs offer superior precision, fit, esthetics, and durability compared to conventional techniques, making them an excellent choice for post-cancer patients requiring customized prosthetic solutions. Additionally, CAD/CAM technology can accelerate the treatment process, which is beneficial for patients who may have difficulty undergoing multiple or prolonged treatment sessions [17,18,19].

For patients with severe post-treatment complications such as trismus and pain, local anesthesia may not be sufficient for extensive dental procedures. General anesthesia (GA) provides a controlled setting that reduces patient discomfort and allows for comprehensive restorative treatment in fewer sessions. The decision to use GA must be considered based on the patient’s overall medical status, with an emphasis on minimizing procedural stress and optimizing rehabilitation outcomes [20].

This article presents a clinical case that highlights the successful application of hospital-based management with advanced technical platforms, including GA and CAD/CAM technology, for a patient with a history of gingivomandibular symphyseal SCC. Given the patient’s history of high-dose radiotherapy, implant placement was deemed high-risk, leading to the selection of a combined approach using fixed and removable prosthetics. This case highlights the importance of conservative management in irradiated patients while using digital tools to enhance prosthetic outcomes. By discussing this case, the article underscores the potential benefits of a multidisciplinary approach to oral rehabilitation, where clinical expertise and technological innovation come together to improve functional and esthetic outcomes for head and neck cancer survivors.

## 2. Case Report

### 2.1. Patient Information

This case report was prepared following the CARE (CAse REport) guidelines [21]. A 58-year-old woman was referred to the special care unit for full-mouth oral rehabilitation in March 2022. This patient presents with a complex medical and oncologic history that impacts her oral health and rehabilitation process. Given her history of advanced gingiva-mandibular symphyseal squamous cell carcinoma, staged as T4aN2c, diagnosed in December 2020 through histopathological examination, she faces multiple post-treatment complications. These include persistent oral pain, eating difficulties, and altered self-perception, all of which require multidisciplinary approach for effective management.

### 2.2. Clinical History and Diagnostic Assessment

Her oncologic treatment included tumor excision in early 2021, followed by 35 sessions of radiotherapy (70 Gy) which started in January 2021 and were completed by February 2021. She also underwent chemotherapy with Cisplatine (50–100 mg/m^2^ intravenously) from 7 January to 22 February 2021. The patient’s medical history included neuropathy with muscle loss, chronic bronchitis, anemia, and bilateral lymphatic edema, which complicates her overall health status. She was an active smoker (10–15 cigarettes/day, 30–40 pack-years) and consumed approximately four alcoholic beverages daily, which contribute to an increased risk of delayed healing, osteoradionecrosis, and poor periodontal outcomes.

**Figure 1:**
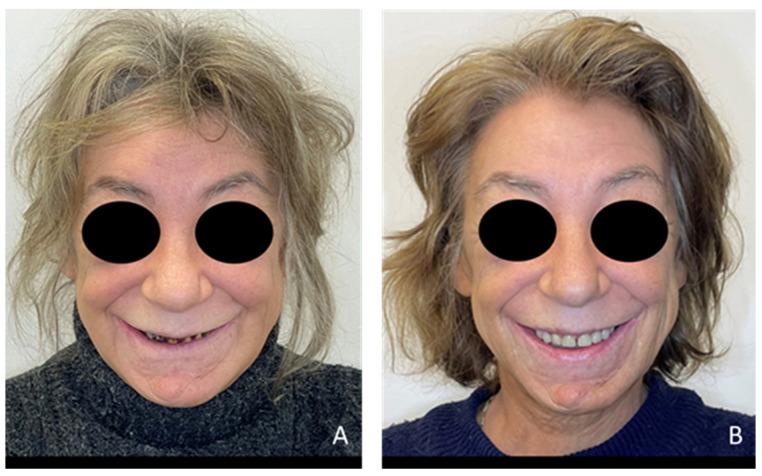
Front View extraoral examination: (**A**) before and (**B**) after treatment.

**Figure 2:**
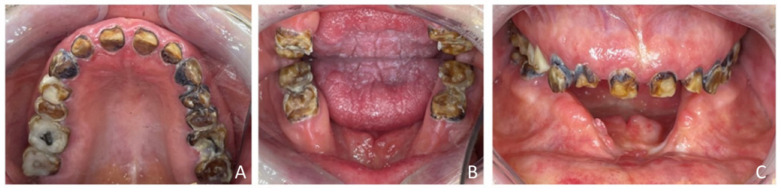
Endo buccal examination: (**A**) maxillary arch, (**B**) mandibular arch, (**C**) dental arches in occlusion.

**Figure 3:**
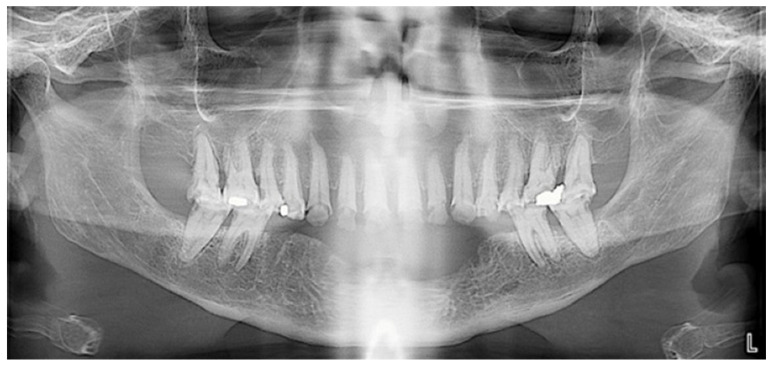
Panoramic radiograph before treatment.

Extraoral examination revealed an unaesthetic smile, radiation-induced caries (Figure 1A), and mandibular retrognathia following carcinoma resection. The occlusal vertical dimension (OVD) was slightly reduced. Intraoral examination showed extensive decay in the remaining teeth (17 to 27, 36, 37, 46, and 47), with most being painful to percussion and highly sensitive to cold. Compromised restorations were noted in teeth 17, 16, 14, and 26 (Figure 2). The gingiva was inflamed and extremely sensitive, and the alveolar ridge in the mandibular incisal region was severely resorbed. Radiographic examination confirmed that 26 and 17 were non-restorable due to advanced root decay and periodontal disease (Figure 3).

### 2.3. Therapeutic Goals

The therapeutic goals were to eliminate infection and pain, improve chewing function, and restore esthetics. The treatment plan included caries removal and root canal treatment for all remaining teeth, except for non-restorable teeth 17 and 26, which were scheduled for extraction. Fiberglass posts and monolithic ceramic crowns were planned for 16, 15, 14, 13, 12, 11, 21, 22, 23, 24, 36, 37, 46, and 47. Tooth 26 was planned to be replaced with a bridge spanning from teeth 25 to 27. Although dental implants have shown promising long-term survival in post-oral cancer patients, the prognosis of implants placed in irradiated bone, particularly when bone augmentation is required, remains compromised. Given the patient’s history of radiation exposure and bone loss, implant therapy was deemed unsuitable to mitigate the risk of implant failure [15]. Instead, a 10-tooth metal-framed removable prosthesis was selected to replace the missing mandibular teeth.

### 2.4. Intervention and Treatment Process

**Figure 4:**
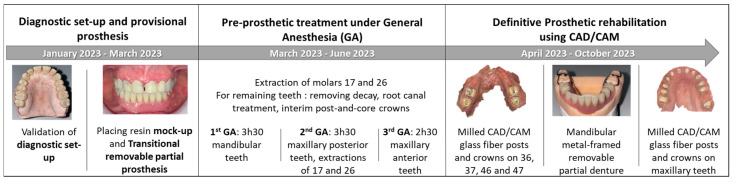
Intervention and steps of treatment process.

The treatment plan was carried out in three steps (Figure 4).

#### 2.4.1. Step 1: Diagnostic Set-Up and Provisional Prosthesis

**Figure 5:**
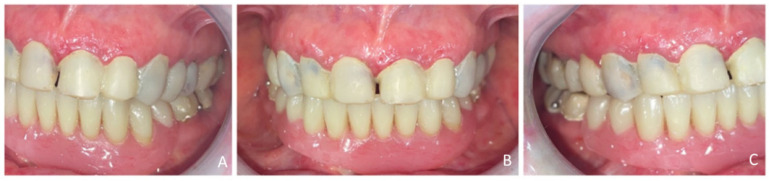
Transitional prosthetic treatment with composite resin mock-up and mandibular transitional partial denture occludes arch’s view: (**A**) right view, (**B**) front view, (**C**) left view.

A diagnostic tooth set-up was created, including a 3 mm increase in occlusal vertical dimension (OVD). It was validated to confirm esthetic and functional outcomes. A transitional removable partial prosthesis (RTPP) was fabricated for the mandible. The OVD was increased using a composite resin mock-up (Voco StructurPremium, Cuxhaven, Germany) applied to all remaining teeth. Adjustments were made to refine the shape of the maxillary anterior teeth. A definitive mandibular impression was taken with polysulfide material (Permlastic, regular body, KaVo Kerr) using a custom tray, and maxillomandibular records were obtained with a wax baseplate. One week later, a 10-tooth RTPP (replacing 45, 44, 43, 42, 41, 31, 32, 33, 34, and 35) was inserted. The provisional prosthetic phase required three clinical sessions (Figure 5).

#### 2.4.2. Step 2: Pre-Prosthetic Treatment Under General Anesthesia

The second step, performed under GA with nasotracheal intubation, was completed in three sessions, totaling 9.5 h. The procedures were organized by sector:

- First GA session (March 2023, lasted 3 h and 30 min): Pre-prosthetic care for mandibular teeth.
- Second GA session (June 2023, lasted 3 h and 30 min): Pre-prosthetic care for maxillary posterior teeth, and non-traumatic extractions of teeth 17 and 26.
- Third GA session (June 2023, lasted 2 h and 30 min): Pre-prosthetic care for maxillary anterior teeth [9].

Each remaining tooth underwent caries debridement, endodontic therapy with rubber dam isolation, root canal preparation, and peripheral tooth preparation for the placement of glass fiber posts and crowns. Interim post-and-core crowns (Voco StructurPremium, Cuxhaven, Germany) were cemented with Temp Bond™ (KaVo Kerr, Brea, CA, USA). To prevent osteoradionecrosis, Amoxicillin with clavulanic acid was prescribed at a dosage of 2 g/day for 15 days, starting before extractions and continuing postoperatively [9].

#### 2.4.3. Step 3: Definitive Prosthetic Rehabilitation

**Figure 6:**
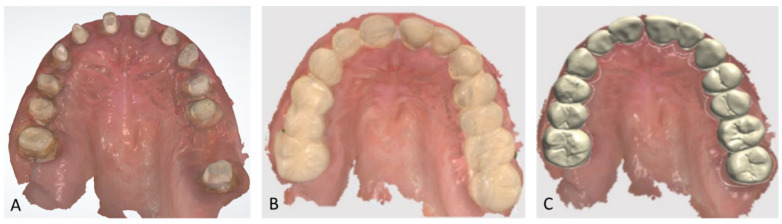
Prosthetic care using CAD/CAM: (**A**) digital cast, (**B**) digital cast with provisional crowns, (**C**) design of crowns.

Definitive prosthetic rehabilitation was performed using computer-aided design and manufacturing (CAD/CAM) technology. The restoration of the mandibular molars (36, 37, 46, and 47) was achieved using milled CAD/CAM glass fiber posts and crowns, followed by the fabrication of a metal-framed removable partial denture and fixed prostheses for the maxillary teeth. Full-arch digital impressions were taken both with and without the provisional crowns using two intraoral scanners: PrimeScan v5.1.3 (Dentsply Sirona, Charlotte, NC, USA) for the fiberglass-reinforced fiber posts (RFPs) and 3Shape v21.2.2 (3Shape A/S, Copenhagen, Denmark) for the crowns (Figure 6). The RFPs were milled from a glass fiber disk (Numerys GF, Itena Clinical, Villepinte, France) and cemented with glass ionomer cement (Fuji 1, GC, Lucerne, Switzerland). The crowns were fabricated from lithium disilicate-reinforced glass-ceramic blocks (Emax, Ivoclar, Amherst, NY, USA) and bonded with light-curing self-adhesive resin cement (Relyx Unicem, 3M, Maplewood, MN, USA) according to the manufacturer’s guidelines.

**Figure 7:**
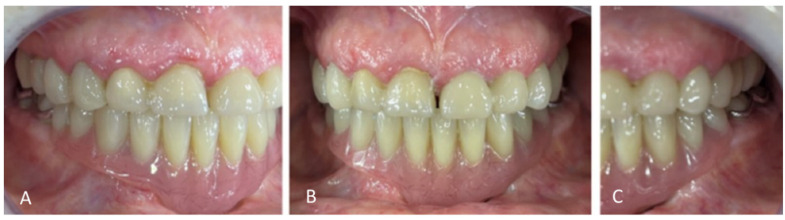
Occluded arches views after treatment: (**A**) right view, (**B**) front view, (**C**) left view.

**Figure 8:**
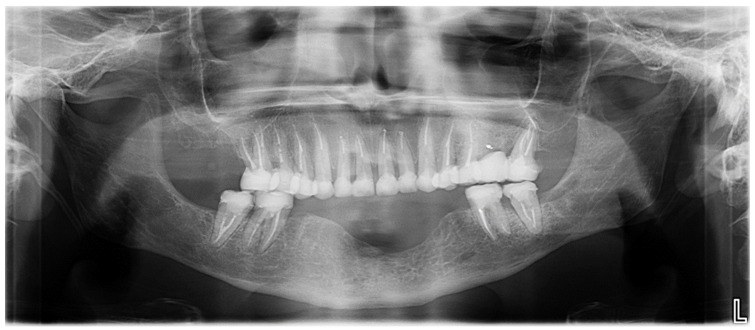
Panoramic radiograph after treatment.

For the mandibular metal-framed removable partial denture, a digital impression was made using the 3Shape intraoral scanner. After the framework try-in, maxillomandibular relationship records were obtained using Aluwax (Aluwax Dental Products Co., Allendale, MI, USA), to accurately mount the casts on the articulator. The set-up was evaluated before polymerization. Upon prosthesis insertion, the occlusion was verified to ensure group function. Figure 1B, Figure 7 and Figure 8 illustrate the final treatment outcome.

### 2.5. Outcomes and Follow-Up

The patient’s health and quality of life were assessed using the University of Washington Quality of Life Questionnaire, version 4 (UW-QOL V4), a validated tool for head and neck cancer patients [22,23]. This tool evaluates multiple dimensions of well-being, including physical health (swallowing, chewing, speech, taste, saliva, and appearance) and socio-emotional aspects (anxiety, mood, pain, activity, leisure, and shoulder function). Each item is scored from 0 to 100, with higher scores indicating a better quality of life. The patient’s scores are presented in Table 1. Following treatment, the patient experienced improvements across multiple domains. Infection control and pain management were successfully achieved, as demonstrated by an increase in the pain score. Esthetic satisfaction improved, with the appearance score rising from 25 to 75.

Oral health-related quality of life was further assessed using the General Oral Health Assessment Index (GOHAI) [24]. This index consists of 12 items grouped into three dimensions: functional (eating, speaking, swallowing), psychosocial (concerns, relational discomfort, appearance), and pain/discomfort (medication use, gingival sensitivity, difficulty chewing certain foods). Each item is rated on a scale from 1 to 5, resulting in a maximum score of 60. Higher scores signify better quality of life: a score of 57–60 indicates satisfactory oral health, 51–56 indicates average quality, and below 50 denotes poor oral health. Before treatment, the patient’s GOHAI score was 30/60, suggesting poor oral health. This increased to 44/60 two weeks after treatment, indicating an improvement in oral function and comfort (Table 2).

Masticatory performance was assessed using chewing gum [25]. This test measures the degree of color mixing in two-colored Hue-Check Gum^®^ (blue and pink) after 20 chewing cycles, assessed on a visual scale [26]. The test revealed a significant improvement following prosthetic rehabilitation. Before rehabilitation, the chewing gum was unmixed (category SA1 on visual scale), indicating poor masticatory performance. After rehabilitation, the expelled gum was classified as SA3, suggesting an average level of masticatory performance. A one-year follow-up appointment was scheduled; however, the patient was unable to attend due to pulmonary complications.

## 3. Discussion

Upon referral for consultation, it was crucial to stop the rapid progression of post-radiation cavities, which were exacerbated by the patient’s pain, and impaired oral hygiene. If left untreated, these lesions could have led to osteonecrosis or total tooth loss [27].

Given the extensive treatment required, GA was selected to facilitate tooth preserving strategy while ensuring patient comfort. The *Haute Autorité de Santé* recommends GA for complex and lengthy procedures, and in this case, three GA sessions were planned to allow for staged interventions with sufficient healing time [28]. Canal preparations and provisional crowns were completed under GA, reducing the number of clinical sessions. Patients with a history of head and neck cancer often experience physical limitations that classify them as individuals with a disability, even if they are willing to cooperate. Extended clinical sessions can be exhausting and demotivating due to fatigue and pain, making GA essential [20,29,30]. GA was necessary in this case due to the challenges encountered with local anesthesia, including ineffective pain control, difficulty maintaining a sealed surgical field, and the patient’s inability to keep the mouth open for prolonged periods. By minimizing the need for extractions, GA supported a conservative treatment approach, which enhanced chewing function and improved the patient’s quality of life [31].

The integration of digital dentistry and CAD-CAM technology significantly improved patient comfort and treatment efficiency. Digital impressions eliminated the discomfort associated with traditional impression materials and allowed for rest periods between procedures. The optical impression of 12 maxillary teeth was successfully captured in a single session, which would have been challenging with conventional methods. CAD-CAM technology further enhanced prosthetic accuracy and workflow efficiency, ensuring precise reproduction of the validated prosthetic design. A preliminary digital impression with a mock-up was created to guide crown fabrication and align the final restoration with the planned outcome.

The patient experienced improvements in both quality of life and oral health following treatment. While masticatory performance improved, as indicated by the chewing gum test, the patient does not fully perceive these benefits; her functional score on the GOHAI remains at 12 out of 20, and the masticatory score on the UW-QoL V4 stands at 50 out of 100, indicating persistent functional challenges. These findings highlight the importance of both objective and subjective masticatory function assessments in evaluating post-treatment outcomes in cancer survivors [32,33]. An implant-supported prosthesis might have further enhanced masticatory performance and overall quality of life [34].

Recent literature primarily focuses on implant-based rehabilitation for post-cancer patients, with CAD/CAM technology highlighted for its precision and efficiency in reducing treatment time [35]. The all-on-four implant protocol, combined with CAD/CAM prostheses, has been documented in oral cancer cases [36]. However, when implant therapy is contraindicated, a removable prosthesis remains the main alternative, often combined with the preservation of residual teeth [15]. For radiotherapy patients, maintaining residual teeth requires a rigorous follow-up every six months, early management of carious lesions with glass ionomer restorations, and strict oral hygiene, including daily fluoride application. In cases of extensive dental damage, extraction is often necessary. In this case, the priority was to preserve the remaining teeth by restoring them with endodontic treatment and a tooth-supported fixed prosthesis rather than opting for extractions.

The originality of this approach lies in the use of a tooth-supported fixed prosthesis to maintain residual dentition, improving both function and esthetics. This treatment was facilitated by GA, which allowed for comprehensive rehabilitation while minimizing patient discomfort. Without GA, the only alternative would have been multiple extractions. Preserving posterior teeth also optimized mastication, contributing to a better functional outcome. This case underscores the importance of a multidisciplinary approach in post-cancer oral rehabilitation. The combination of fixed and removable prosthetics successfully restored function and esthetics. GA enabled efficient treatment sequencing, minimizing clinical sessions while ensuring patient comfort. Additionally, CAD/CAM technology enhanced prosthetic accuracy and streamlined workflows, demonstrating the benefits of digital dentistry in complex cases.

Clinically, this case report reinforces the value of personalized, conservative strategies over conventional extractions, particularly for medically complex patients. It highlights the role of GA in facilitating extensive dental rehabilitation and the advantages of CAD/CAM in improving prosthetic outcomes. By documenting this case, we provide a framework for clinicians managing head and neck cancer survivors, emphasizing a multidisciplinary approach to optimize both function and quality of life.

This case is limited by its short follow-up period, which may not assess long-term functional outcomes or complications related to oral rehabilitation following squamous cell carcinoma treatment. As a case report, it describes a single patient’s experience, which limits the generalizability of the findings to larger populations. Case reports occupy a lower position in the hierarchy of clinical evidence due to their descriptive nature and lack of statistical power. To strengthen the evidence base, future research should aim to assess the long-term outcomes of prosthetic rehabilitation and its impact on patients’ quality of life through larger, controlled, and prospective studies.

## 4. Conclusions

This case highlights the advantages of a multidisciplinary and conservative approach in rehabilitating head and neck cancer survivors. The integration of GA and CAD/CAM technology facilitated the preservation of residual dentition, improved prosthetic outcomes, and enhanced both function and esthetics. Future research should focus on evaluating the long-term success of prosthetic rehabilitation and their impact on quality of life.

## Figures and Tables

**Table 1:** UW-QOL scores before and after treatment compared to reference values.

UW-QOL Domains	Scores Prior to Treatment (/100)	Scores After Treatment (/100)	Healthy Population Reference Scores (/100)
Chewing	50	50	94
Swallowing	100	100	98
Speech	70	70	98
Taste	100	100	95
Saliva	30	100	97
Appearance	25	75	93
Physical score	62.5	82.5	95.8
Anxiety	70	70	83
Mood	75	75	82
Pain	25	75	86
Activity	50	50	86
Recreation	75	75	86
Shoulder	100	100	91
Socio-emotional score	65.9	74.2	85.7
Global score	64.2	78.35	90.8

**Table 2:** Changes in GOHAI scores before and after treatment.

GOHAI Domains	Scores Prior to Treatment	Scores After Treatment
Functional domain (/20)	12	12
Psychosocial domain (/25)	14	22
Pain and discomfort (/15)	4	10
Total score (/60)	30	44

## Data Availability

Data are contained within the article.
